# Defining optimal implementation packages for delivering community-wide mass drug administration for soil-transmitted helminths with high coverage

**DOI:** 10.1186/s12913-022-08080-5

**Published:** 2022-06-18

**Authors:** Marie-Claire Gwayi-Chore, Kumudha Aruldas, Euripide Avokpaho, Chawanangwa Maherebe Chirambo, Saravanakumar Puthupalayam Kaliappan, Parfait Houngbégnon, Comlanvi Innocent Togbevi, Félicien Chabi, Providence Nindi, James Simwanza, Jabaselvi Johnson, Edward J. Miech, Khumbo Kalua, Moudachirou Ibikounlé, Sitara S. R. Ajjampur, Bryan J. Weiner, Judd L. Walson, Arianna Rubin Means

**Affiliations:** 1 Department of Global Health, University of Washington, Seattle, USA; 2 The DeWorm3 Project, University of Washington, Seattle, WA USA; 3 The Wellcome Trust Research Laboratory, Division of Gastrointestinal Sciences, Christian Medical College, Vellore, India; 4 Institut de Recherche Clinique du Benin, Abomey-Calavi, Benin; 5 Blantyre Institute for Community Outreach (BICO), Lions Sight First Eye Hospital, Blantyre, Malawi; 6 Center for Health Services Research, Regenstrief Institute, Indianapolis, USA; 7 Kamuzu University of Health Sciences, Blantyre, Malawi; 8 Centre de Recherche pour la Lutte Contre les Maladies Infectieuses Tropicales, Université d’Abomey-Calavi, Abomey-Calavi, Bénin; 9 Departments of Medicine, Pediatrics, & Epidemiology, University of Washington, Seattle, USA

**Keywords:** Coincidence analysis, Configurational comparative methods, Neglected tropical disease, Global health implementation science

## Abstract

**Background:**

Recent evidence suggests that community-wide mass drug administration (MDA) may interrupt the transmission of soil-transmitted helminths (STH), a group of intestinal worms that infect 1.5 billion individuals globally. Although current operational guidelines provide best practices for effective MDA delivery, they do not describe which activities are most essential for achieving high coverage or how they work together to produce effective intervention delivery. We aimed to identify the various packages of influential intervention delivery activities that result in high coverage of community-wide MDA for STH in Benin, India, and Malawi.

**Methods:**

We applied coincidence analysis (CNA), a novel cross-case analytical method, to process mapping data as part of the implementation science research of the DeWorm3 Project, a Hybrid Type 1 cluster randomized controlled trial assessing the feasibility of interrupting the transmission of STH using bi-annual community-wide MDA in Benin, India, and Malawi. Our analysis aimed to identify any necessary and/or sufficient combinations of intervention delivery activities (i.e., implementation pathways) that resulted in high MDA coverage. Activities were related to drug supply chain, implementer training, community sensitization strategy, intervention duration, and implementation context. We used pooled implementation data from three sites and six intervention rounds, with study clusters serving as analytical cases (*N* = 360). Secondary analyses assessed differences in pathways across sites and over intervention rounds.

**Results:**

Across all three sites and six intervention rounds, efficient duration of MDA delivery (within ten days) singularly emerged as a common and fundamental component for achieving high MDA coverage when combined with other particular activities, including a conducive implementation context, early arrival of albendazole before the planned start of MDA, or a flexible community sensitization strategy. No individual activity proved sufficient by itself for producing high MDA coverage. We observed four possible overall models that could explain effective MDA delivery strategies, all which included efficient duration of MDA delivery as an integral component.

**Conclusion:**

Efficient duration of MDA delivery uniquely stood out as a highly influential implementation activity for producing high coverage of community-wide MDA for STH. Effective MDA delivery can be achieved with flexible implementation strategies that include various combinations of influential intervention components.

**Supplementary Information:**

The online version contains supplementary material available at 10.1186/s12913-022-08080-5.

## Background

Policymakers and implementers are often challenged with making decisions regarding the implementation of complex interventions to ensure high coverage and uptake [1–3]. Complex interventions, by definition, have numerous interrelated elements that impact both implementer processes and recipient responses, including their intervention components, implementation strategy, and contextual features [4, 5]. Therefore, there is a critical need for approaches that evaluate the relationships between these elements and determine their influence on effective intervention delivery in order to identify under what circumstances complex interventions are successful [1, 6, 7]. Outcomes from these assessments can support evidence-based decision-making for policymakers and implementers, especially those working within health systems in low- and middle-income countries (LMICs) who often face issues of limited resources and capacity [8–11].

One such complex intervention is mass drug administration (MDA) for neglected tropical diseases (NTDs) – a group of parasitic, viral, and bacterial diseases that affect billions of individuals, with disproportional prevalence in LMICs across sub-Saharan Africa, South Asia, and Latin America [12, 13]. Untreated NTD infections negatively impact health, learning and productivity outcomes, diminishing quality-of-life and reinforcing cycles of poverty amongst the world’s most disadvantaged populations [12, 13]. Worldwide, the most prevalent NTDs are soil-transmitted helminths (STHs), a group of parasitic intestinal worms that infect approximately 1.5 billion people globally – of these, an estimated 900 million are pre-school and school-age children [13–15]. Current World Health Organization (WHO) guidelines recommend geographic areas that meet pre-defined STH prevalence thresholds to implement MDA, with a particular focus on deworming pre-school and school-age children in schools. In these programs, all at-risk children receive anti-helminthics, such as albendazole, regardless of their infection status [12, 13]. This school-based MDA approach is the standard-of-care for STH control across numerous LMICs, reaching hundreds of millions of children annually [13, 14, 16]. However, this approach does not target adults who serve as infection reservoirs in communities, thus contributing to rapid pediatric reinfection [12, 16]. A community-wide MDA approach that targets community members of all ages shows promise of interrupting the transmission of STH [17–19]. However, even with this intensified treatment strategy, MDA must be delivered with high coverage, with at least 80–90% of the targeted population dewormed, to achieve transmission interruption [17–21].

Significant evidence suggests best practices for delivery of MDA with high treatment coverage include: a reliable drug supply chain to ensure the adequate allocation and distribution of anthelmintic drugs; a robust training cascade to build implementer capacity; a far-reaching community sensitization strategy to inform and mobilize recipients about MDA; and a well-executed, yet rapid distribution strategy [15, 22–29]. Thus, effective MDA delivery requires a significant investment of material, financial, and human resources as well as adaptable implementation strategies that are feasible, appropriate, and acceptable for heterogeneous implementation settings. However, current MDA operational guidelines issued by the WHO and national Ministries of Health in NTD-endemic countries lack context-specific recommendations that take into account variation in key implementation factors, such as disease epidemiological profiles, community preferences, and health system capacities [16, 23, 24, 28, 30]. Furthermore, guidelines do not distinguish which implementation activities are most essential for achieving MDA delivery with high coverage. This information is necessary for implementers who are planning MDA at scale in resource constrained environments, and who may not be able to incorporate all best practices into an implementation plan.

Using coincidence analysis (CNA), a cross-case analytical method, we systematically identify the various configurations of intervention delivery activities – known as implementation pathways – that result in high coverage of community-wide MDA for STH. This analysis aimed to characterize the “core components” of MDA delivery – activities that are necessary for achieving high coverage and need to be implemented with fidelity [31]. Such evidence may help policymakers define the required resources for implementing MDA with high coverage as well as shape implementer decisions regarding implementation that balances fidelity with flexibility.

## Methods

### Methodological background

CNA is a type of configurational comparative method (CCM) based on Boolean algebra that determines specific combinations of conditions called configurations whose presence or absence “makes a difference” (i.e. are difference-makers) as to whether an outcome of interest occurs [32, 33]. CCMs are based on regularity theories of causation that utilize cross-case comparisons to identify these difference-makers. Thus, in comparison to regression-based analyses, these approaches fundamentally identify different properties of causal structures [32, 34]. Of particular utility is the ability to model Boolean conjunctivity, where multiple conditions must be jointly present to bring about an outcome, and equifinality, where multiple pathways lead to the same outcome [32, 34]. These two principles are of particular interest to implementers and policymakers, as interventions delivered in real-world settings are often complex and feature numerous interdependent activities that can produce various outcomes based on the selected implementation strategy [35].

CNA enable the complex modeling of the relationships between influential factors that cannot be captured with traditional statistical approaches [32, 34]; thus, it is gaining traction as an additional approach for evaluating program implementation [35–40]. The aim of CNA is to identify the minimal set of necessary and/or sufficient configurations to achieve an outcome of interest [32, 33]. A necessary condition/configuration must be present for the outcome to occur (but does not produce the outcome by itself), while a sufficient condition/configuration can produce the outcome alone [41, 42]. Certain conditions, called “INUS” conditions, are neither sufficient nor necessary alone, but as part of a configuration, play an influential role in producing the outcome of interest. INUS represents a condition that is Insufficient (not sufficient by itself to produce an outcome) but a Necessary component of a configuration that is itself Unnecessary (due to multiple pathways) but Sufficient for the outcome to occur [41, 42]. In real-world implementation, it is rare that any single program activity produces the outcome of interest; thus identifying these INUS conditions is especially relevant for evaluating intervention delivery [34].

### Study background & setting

We applied CNA to process mapping data from the DeWorm3 Project, a Hybrid Type 1 cluster randomized controlled trial conducted in three sites – Benin, India, and Malawi – that aim to assess the feasibility of interrupting the transmission of STH, defined as weighted cluster-level prevalence < 2% measured 24 months after the final round of MDA [43, 44]. The primary study objective is to evaluate the impact of bi-annual community-wide MDA as compared to school-based MDA on STH infection prevalence [43]. Over three consecutive years, 40 clusters per site, which consisted of one or more administrative villages, settlements or zones, were randomized to receive bi-annual community-wide MDA delivered by trained community drug distributors (CDDs) or school-based MDA delivered by trained teachers, in accordance with WHO recommendations and national Ministry of Health guidelines [43]. Embedded in the trial is a robust implementation science research component – including stakeholder mapping, qualitative research, structural readiness assessments, process mapping, and economic evaluation methods – that aims to contextualize trial findings, optimize intervention delivery, and identify strategies for successful intervention scale-up [44].

*Sampling & Data Collection.*

Data from this analysis comes from the process mapping component of DeWorm3 implementation science research, which details the required inputs for effective intervention delivery [44]. During each round of MDA, routine process mapping exercises were conducted in each intervention cluster. Trained implementation science research staff tracked the completion and timing of key intervention delivery activities in five domains: *drug supply chain* (e.g. quantity and timing of albendazole arrival), *training* (e.g. timing and proportion of implementers trained prior to delivery), *community sensitization* (e.g. type of community sensitization activities conducted), *intervention duration* (e.g. number of days of MDA delivery), and *implementation context* (e.g. presence of ongoing community interventions or events that may have impacted MDA delivery). Data were recorded on paper copies of standardized process mapping worksheets (Supplementary Materials) and subsequently entered into the DeWorm3 SurveyCTO database [45].

### Measures & analysis

Our analytical process was adapted from Whitaker et al. [35]. Intervention clusters served as analytical cases. Our primary analysis aimed to identify configurations that led to high coverage of community-wide MDA across all sites and intervention rounds using one pooled dataset including 360 cases (20 intervention clusters across 6 MDA rounds for three sites). The primary outcome of interest was high coverage (> 90%) of community-wide MDA, as per trial protocol [43]. The initial dataset included 11 possible factors based on the 5 MDA implementation domains included in the process mapping exercise (Table 1). Each factor was previously identified as a key variable that impacts effective delivery of community-wide MDA for various NTDs [15, 22–27, 29, 46]. With configurational approaches, factors with insufficient variability cannot be difference-makers as they do not distinguish cases with and without the outcome [32]. In order to make this determination, we used plots to visualize the distribution of all 11 factors, removing two forms of mass media sensitization strategies (newspaper and television) that had very low (< 1%) variation, leaving nine factors suitable for further analysis. In CNA, conditions are the specific values a factor takes on based on data calibration choice. We used crisp-set (i.e. dichotomous) calibration for the outcome and all conditions as it is straightforward to interpret and operationally actionable as compared to other calibration choices [47].

**Table 1:** Description and calibration of CNA variables

Outcome/Condition	Description	Factor	Calibration
High coverage of community-wide MDA	> 90% (per-protocol) ^1^	COVERAGE	0 < 90% coverage 1 > 90% coverage
Drug supply chain	Timing of when albendazole arrive in each cluster	DRUG	0 Late arrival of albendazole (on first day of MDA) 1 Early arrival of albendazole (at least one day before MDA)
Implementer training	Proportion of community drug distributors (CDDs) trained prior to MDA in cluster	TRAIN	0 Not all CDDs trained 1 All CDDs trained
Community sensitization type	Type of community sensitization activity conducted in cluster ^2^	MTG, PUBLIC, PRINT, DOOR, RADIO, TV, NEWSPAPER	0 Sensitization activity not conducted 1 Sensitization activity conducted
Intervention duration	Number of days of MDA delivery	MDADAYS^4^	0 MDA delivered in > 10 days 1 MDA delivered in <= 10 days
Implementation context	Presence of ongoing interventions or events in the community that may have negatively impacted MDA delivery or uptake	CONTEXT	0 Non-conducive implementation context (at least one ongoing community intervention or event) 1 Conducive implementation context (no community interventions or events)

^1^Per-protocol coverage defined as: the percentage of censused and eligible individuals treated with a single dose of albendazole.

^2^Sensitization activities incudes: community meetings (MTG), public address announcements (PUBLIC), distribution of printed education materials e.g., posters and banners (PRINT), door-to-door sensitization (DOOR), or mass media (RADIO, TV, NEWSPAPER).

^3^Television (TV) and news (NEWSPAPER) were removed from final dataset due to limited variation across cases.

^4^CDDs who participated in DeWorm3 were given 10 days to deliver community-wide MDA.

CNA models have two overall measures of fit: consistency and coverage. Consistency indicates the “reliability” of a CNA model by measuring the number of cases identified by the overall model with the outcome of interest over the total number of all cases identified by the overall model [32, 41, 42, 47]. Coverage is an indicator of the “relevance” of a model by measuring the number of cases identified by the overall model with the outcome of interest (i.e., the same numerator as for consistency) over all cases with the outcome present [32, 41, 42, 47]. Consider an analysis conducted within a hypothetical dataset of 100 high-coverage clusters and 100 low-coverage clusters. If 80 clusters across the dataset had the same configurations, and 75 of these 80 had the outcome present, the overall consistency and coverage scores for this model would be 0.94 (75/80) for consistency and 0.75 (75/100) for coverage. Depending on the dataset and the thresholds set for consistency and coverage, CNA may yield several candidate models that fit the data equally well (i.e., similar consistency and coverage scores), a situation known as model ambiguity [35, 48]. In this scenario, it is not possible on mathematical grounds alone to select one model as the “correct” one; rather, theory, background knowledge, and case familiarity may need to be called upon in order to choose one model over the others. Another strategy when faced with model ambiguity is to identify common elements that appear across all model possibilities. Even if it cannot be absolutely determined which single model is the correct one, if all candidate models contain the same identical component, then it follows that this component directly relates to the outcome of interest.

Using a data reduction approach described previously in the configurational literature [38, 39, 48], we first aimed to select the most influential factors to include in iterative model development and analysis. We used the “minimally sufficient conditions” (*msc*) function in the R *cna* package [49] to simultaneously consider all nine factors and 360 cases at once to identify configurations with the strongest relationship to the outcome, as measured by coverage scores. We set the initial consistency threshold at 1.0, reducing it by increments of 0.05 until we observed configurations at the specified threshold. We then ranked these configurations by coverage score, considering all one-, two- and three-condition configurations with the highest coverage scores and aligned with theory, background knowledge and case familiarity.

With this subset of factors, we then iteratively developed models using the R *cna* package [49]. We selected a final model with consistency and coverage score thresholds of > 0.85 and > 0.50, respectively, without model ambiguity. Model interpretation followed conventional Boolean analysis with conditions in uppercase/lowercase representing the presence/absence of a condition, the asterisk “*” symbolizing the logical operator *AND* (i.e., a conjunct), plus sign “**+**” symbolizing the logical operator *OR* (i.e., a disjunct), and the one sided-arrow “→” expressing sufficiency [35, 50].

Our primary analysis aimed to identify configurations – or implementation pathways – that led to high MDA coverage across all three sites and six intervention rounds. This analysis was conducted with one pooled implementation dataset including 360 cases (20 intervention clusters across six MDA rounds for three sites). We also conducted two secondary analyses to assess differences in pathways: a cross-site analysis to consider contextual variation in implementation across countries, and a longitudinal analysis to evaluate potential changes in pathways over MDA rounds. The longitudinal analysis was conducted using six MDA round-level datasets, each including 60 cases (20 intervention clusters for three sites) and the cross-site analysis was conducted using three site-level datasets, each including 120 cases (20 intervention clusters over six MDA rounds). All analyses were performed using RStudio v.1.3.959 using the *cna* package [49].

## Results

### Descriptive statistics

**Fig. 1:**
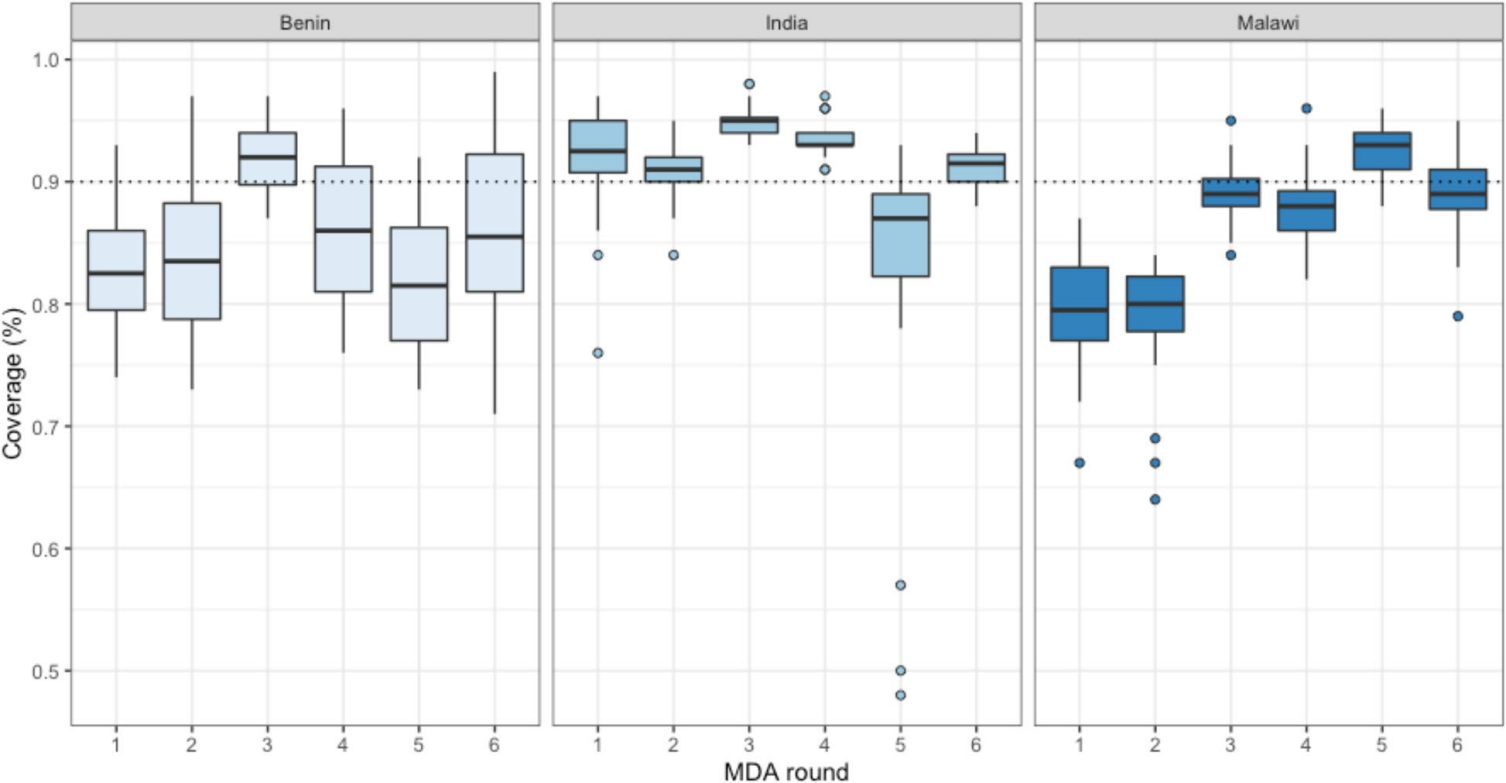
Median community-wide MDA coverage rates, by site and intervention round

High MDA coverage was achieved 171 times across all six intervention rounds – 94 times (55%) in India, 39 times (23%) in Malawi, and 38 times (22%) in Benin (Table 2). Across MDA rounds, round three had the highest number of clusters (*n* = 43) that achieved high coverage across sites, while round one had the lowest (*n* = 17). Across sites, India had the highest median MDA coverage in nearly all MDA rounds (Fig. 1*, Median community-wide MDA coverage rates, by site and intervention round*). Across all sites and over six rounds, the median number of days of MDA delivery was 11 days (range 4–18 days).

**Table 2:** Number of clusters that achieved high MDA coverage, by site and MDA round

Site	Round 1	Round 2	Round 3	Round 4	Round 5	Round 6	All Rounds
Benin	1	4	15	7	3	8	***38***
India	16	16	20	20	4	18	***94***
Malawi	0	0	8	5	18	8	***39***
*All Sites*	***17***	***20***	***43***	***32***	***25***	***34***	***171***

### CNA analysis

The data reduction process identified seven factors to use in subsequent model development at 0.80–0.50 consistency-coverage score thresholds: drug supply chain (DRUG), printed sensitization materials (PRINT), intervention duration (MDADAYS), door-to-door sensitization (DOOR), public address announcements (PUBLIC), community meetings (MTG), and implementation context (CONTEXT). No single condition alone was sufficient for achieving high MDA coverage. Our analysis revealed four possible models sufficient for attaining high MDA coverage:

1. MDADAYS*PRINT + MDADAYS*PUBLIC*CONTEXT → COVERAGE
2. MDADAYS*DRUG + MDADAYS*PRINT → COVERAGE
3. MDADAYS*DRUG + MDADAYS*PUBLIC*CONTEXT → COVERAGE
4. MDADAYS*PRINT + MDADAYS*PUBLIC*DOOR → COVERAGE

For clarification, we include a plain-language interpretation for Model 1. In this model, there are two pathways for clusters to achieve high MDA coverage: (a) PRINT*MDADAYS: distribution of printed health education materials *AND* delivery of MDA within 10 days *OR* (b) PUBLIC*CONTEXT*MDADAYS: public address announcements *AND* a conducive implementation context *AND* delivery of MDA within 10 days.

**Fig. 2:**
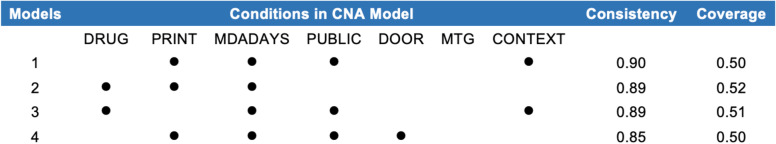
Conditions represented within CNA models for achieving 228 high coverage of community-wide MDA for STH

Note that across the four possible models, there are only a total of four specific configurations represented; each candidate model is a disjunct of those configurations. Although the similar consistency and coverage scores across models (Fig. 2, *Conditions represented within CNA models for achieving high coverage of community-wide MDA for STH*) indicate model ambiguity, the analysis successfully identified one INUS condition – MDADAYS (efficient MDA delivery duration) – which was observed not only across all four possible models, but was part of every configuration within those four solutions. Although MDADAYS alone was insufficient for the outcome to occur, it was a necessary component of each of the four observed pathways across the models: (a) PRINT*MDADAYS, (b) DRUG*MDADAYS, (c) PUBLIC*CONTEXT*MDADAYS, and (d) PUBLIC*DOOR*MDADAYS). Although the cross-site and longitudinal analyses revealed several possible models, they did not meet the pre-specified analytical consistency and coverage score thresholds and were not reported.

## Discussion

This analysis indicates that, while insufficient or necessary by itself, efficient duration of MDA delivery (within 10 days) in combination with at least one other influential implementation activity – including a conducive implementation context, early arrival of albendazole before the start of MDA, or a flexible community sensitization strategy – consistently led to high coverage of community-wide MDA for STH.

Thus, our results suggest that efficient MDA delivery duration was one of the most influential implementation activities for producing high treatment coverage and MDA delivery within 10 days appears to be an optimal delivery timeframe for community-wide MDA for STH. Current evidence shows that the number of scheduled MDA days plays a significant role in the ability of implementers to meet necessary MDA targets, with overly brief durations negatively impacting treatment coverage [23, 51–53]. However, these studies do not determine a definitive timeframe for effective delivery. To our knowledge, this is the first study to assess the effectiveness of a specific duration of MDA delivery.

Our findings mark an important addition to the NTD evidence base, as MDA duration has several implications for policy makers, NTD program managers, and implementers. Primarily, decision-making regarding program duration affects financial and material resources allocated for implementation. Thus, having clarity around the adequacy of scheduled MDA duration may support implementers to more effectively plan for MDA and maintain cost-effectiveness of the intervention, which is a key policy consideration for transitioning to community-wide MDA for STH [54, 55]. Specific to planning, it is critical that implementers allocate a sufficient number CDDs for the target population size for the given campaign delivery schedule [23, 56]. Notably, there may be an important relationship between campaign duration and size of the available workforce; a larger number of CDDs may be necessary to deliver MDA over a shorter duration of time as compared to the number of CDDs needed to deliver MDA to the same target population over a longer period of time. While these results do not directly call for implementers to deliver MDA faster than necessary, they do provide additional consideration for the potential diminishing returns if the number of days are over-extended, given the additional financial and opportunity costs of community-wide MDA as compared to school-based MDA [54, 57]. These findings also provide novel evidence for an intervention component that is not currently highlighted in NTD operational manuals. A potential area of future CNA research could further examine what specific factors distinguish areas that deliver MDA more efficiently to further strengthen these findings.

Although were ultimately unable to single out a single model to explain high MDA coverage due to model ambiguity, the pathways within the candidate models demonstrate how high MDA coverage could potentially be accomplished in various ways, reflecting the utility of flexible implementation strategies, especially for sensitization strategies. Our results indicate that in addition to efficient MDA duration, three components – community sensitization, drug supply chain, and implementation context – consistently appeared across the models, suggesting their influence in achieving high MDA coverage. These three components are heavily outlined in the existing evidence base as key factors that influence the delivery of and demand for community-wide MDA for various NTDs, as summarized below.

### Community sensitization

It is critical that community members are aware of upcoming MDA campaigns and have trust in their efficacy and safety. Sensitization helps build awareness, demand, and trust amongst recipient community members and also builds buy-in from key political, civic, and community stakeholders [24, 25]. Numerous studies have demonstrated the importance of well-designed, multifaceted community awareness strategies on MDA coverage [25, 27, 51, 56, 58–60]. Our findings further demonstrate the strong influence of various sensitization approaches and indicate the importance of flexibility when designing sensitization strategies – in some areas, distribution of written materials may be more effective, while in other areas door-to-door sensitization may be more appropriate.

### Drug supply chain

Another critical component of STH MDA delivery is having sufficient amounts of deworming drugs. Shortage of drugs is a potential consequence of late drug arrival, as the delay forces implementers to rely on stocks of deworming drugs in local health clinics to initiate MDA, which are likely in inadequate amounts to reach all targeted populations. Thus, timely drug arrival is an indication of adequate planning as well as a functional supply chain, which are both predictors of MDA coverage [61–63]. Therefore, late drug arrival often serves as a barrier to achieving high MDA coverage [61–63]. Our results further illustrate how timely drug arrival serves as a facilitator for effective MDA delivery. These findings define the importance of effective planning to mitigate supply chain issues and the need for robust supply chain management to help ensure timely and adequate receipt of necessary drugs.

### Implementation context

The implementation context plays a significant role in intervention delivery [2, 4, 5]. There are a number of contextual challenges that may impede MDA implementation and result in fewer individuals being dewormed. In our study, clusters identified several contextual issues that affected MDA delivery, including other ongoing community health programs, heavy rainy seasons, local cultural festivals and religious events. Our analysis suggests that clusters without these contextual challenges positively influenced MDA coverage. Existing evidence highlights the importance of scheduling MDA around other community events that may negatively affect treatment coverage, or reduce the availability of implementers to deliver MDA, including ongoing public health priorities and programs, important religious or cultural events, community activities, or weather periods [23, 56]. Our findings emphasize the importance of careful planning and cross-sectorial collaboration and communication that in turn, could increase the likelihood of an optimal implementation context.

Overall, our findings illustrate important findings for inclusion in future operational guidelines for delivering community-wide MDA for STH and other NTDs. Policymakers and national-level NTD program managers can utilize these findings when developing MDA budgets and implementation strategies to ensure there are sufficient financial, human, and material resources. Additionally, implementers can use these findings to ensure they sufficiently invest in intervention planning before intervention delivery.

Our analysis has several strengths. Primarily, we included a large amount of implementation data covering six intervention delivery rounds and three distinct geographic settings. These data were pulled from a multi-country study that followed a comprehensive data collection process. We also applied a rigorous analytical process involving the use of CNA within implementation science [35]. However, these results have several limitations. Primary is our use of data from an ongoing hybrid trial; therefore, our findings may not be completely generalizable to MDA programs implemented under routine practice. Other limitations of generalizability are specific to the CNA methodology. Specifically, our results are directly affected by our calibration choices and might have differed with other calibration thresholds. Additionally, although we were able to identify key operational variables that influence MDA coverage, there were other contextual factors that could have impacted intervention delivery that were not included as part of our analysis, including: degree of community trust or acceptability towards the intervention, implementer satisfaction and motivation (e.g. with workload or incentives), and level of community migration over time [24]. The candidate models we reported each had coverage scores around 0.50, indicating a role for other factors and additional pathways to successful intervention delivery. The influence of these factors will be assessed in other planned DeWorm3 coverage analyses. Despite these limitations, we further demonstrated the utility of CNA in modeling complex causality for complex intervention delivery. Thus, our results have substantive implications for the future implementation of community-wide MDA to interrupt the transmission of STH across various low-resource settings.

It is also important to note the impact of the COVID-19 pandemic on study implementation activities. Sites were under country-wide lockdown during a significant portion of the last year of planned MDA delivery (i.e., MDA rounds 5 and 6), and all MDA planning and delivery activities were suspended until as late as July 2020. When field activities resumed, sites captured the impact of the pandemic on MDA implementation on the routine process mapping worksheets, e.g., noting how the pandemic negatively impacted the implementation context in some clusters. Thus, this analysis does capture some of these pandemic-specific challenges. However, we acknowledge the need for additional quantitative and qualitative research that directly assesses the impact of the COVID-19 pandemic on MDA planning and delivery and subsequent transmission interruption progress [64]. Currently, there are several ongoing DeWorm3 evaluations assessing these impacts and understanding how the study’s existing community-wide infrastructure could be utilized to facilitate rapid community responses to COVID-19.

## Conclusion

Using an innovative analytic approach, we identified that efficient duration of MDA delivery within 10 days was a highly influential implementation activity for achieving high coverage of community-wide MDA when co-implemented with other key implementation factors such as a conducive implementation context, early arrival of albendazole, and a flexible community sensitization strategy. These findings can be used by STH-endemic countries implementing MDA programs to develop appropriate operational guidelines and support effective implementation planning. Similar methodological approaches may be extended to evaluate other community-based primary care programs implemented in LMIC health systems.

## Supplementary Information

**Additional file 1.**

## Data Availability

The datasets used and/or analyzed during the current study are available from the corresponding author on reasonable request.

## Abbreviations

MDA: Mass drug administration
NTD: Neglected tropical disease
STH: Soil-transmitted helminths
CNA: Coincidence analysis
LMIC: Low- and-middle-income country
WHO: World Health Organization
