# Supplementary material for: Defining optimal implementation packages for delivering community-wide mass drug administration for soil-transmitted helminths with high coverage
Source: BMC Health Serv Res. 2022 Jun 18;22:792. doi: 10.1186/s12913-022-08080-5 (PMC9206125; doi:10.1186/s12913-022-08080-5)
Supplement: Supplementary file 1 — Additional file 1. [file 12913_2022_8080_MOESM1_ESM.pdf]

deworm3

Routine Workflow Tracking Worksheet: Community-Wide MDA

Country:_______________ **MDA Round:** ____ **Cluster ID** _________ **Submission Date**: _______________

Instructions: Please complete this form for every cluster that delivers community-wide MDA (20 clusters).

**Activity**		**Completion Data**	
**Drug Supply Chain**			
Date albendazole arrived in cluster		____- ____ -______<br>*DD-MM-YYYY*	
First day that community drug distributors (CDDs) collected albendazole and total number of tablets collected		____- ____ -______<br>*DD-MM-YYYY*	______ *tablets*
Number of days of albendazole collection by CDDs		___________ *days*	
Number of tablets returned		__________ *tablets*	
**CDD Training**			
Number of CDDs trained		__________/ __________<br>(*No. CDDs trained/Total no. of CDDs)*	
Date of CDD training<br>*If training occurred on more than one day, write all dates*		____- ____ -______<br>*DD-MM-YYYY*<br>____- ____ -______<br>*DD-MM-YYYY*<br>____- ____ -______<br>*DD-MM-YYYY*	____- ____ -______<br>*DD-MM-YYYY*<br>____- ____ -______<br>*DD-MM-YYYY*<br>____- ____ -______<br>*DD-MM-YYYY*
**Community Sensitization**			
For each sensitization activity:<br>1. Indicate which activities occurred in the cluster prior to this round of MDA (tick all that apply)<br>2. Indicate the quantity of the sensitization activity<br>3. Indicate the date when the activity started in the cluster	☐ Community meetings	__________<br>*No. of meetings*	____- ____ -______<br>*DD-MM-YYYY*
	☐ Public dialogue event<br>*(e.g. drama or town crier)*	__________<br>*No. of events*	____- ____ -______<br>*DD-MM-YYYY*
	☐ Distribution of printed IEC materials<br>*(e.g. pamphlets or posters)*	__________<br>*No. distributed*	____- ____ -______<br>*DD-MM-YYYY*
	☐ Door-to-door sensitization	__________<br>*No. of houses visited*	____- ____ -______<br>*DD-MM-YYYY*
	☐ Mass media (circle one):<br>Radio TV Newspaper Other	__________<br>*No. of media spots*	____- ____ -______<br>*DD-MM-YYYY*
	☐ Other (please specify):<br>____________________________	__________<br>*Specify no.*	____- ____ -______<br>*DD-MM-YYYY*
**MDA Delivery**			
Dates of first and last days of MDA		____- ____ -_____TO ____- ____ -_____<br>*DD-MM-YYYY* *DD-MM-YYYY*	
Dates of first and last days of MDA mop-up		____- ____ -_____TO ____- ____ -_____<br>*DD-MM-YYYY* *DD-MM-YYYY*	
**Monitoring & Evaluation**			
Please note any community activity that took place in the cluster one month prior to first day of MDA that may have affected treatment coverage. Include the date of the first and last days of the activity (DD-MM-YYYY).<br>*(If you need more space, please write on the back of this page)*			

Please write your name on this worksheet to confirm accuracy of data:

Implementation Science Supervisor: ______________________________

Cluster Lead ________________________________
